# Educational exposures associated with preclinical medical student interest in pursuing surgical residency: Longitudinal mixed-methods study with narrative evaluation

**DOI:** 10.1016/j.sopen.2023.03.002

**Published:** 2023-03-03

**Authors:** Adree Khondker, Michael Ho-Yan Lee, Emilia Kangasjarvi, Jory S. Simpson

**Affiliations:** a Temerty Faculty of Medicine, University of Toronto, Toronto, Ontario, Canada; b Li Ka Shing Knowledge Institute, Applied Education Research Operatives, Faculty of Medicine, University of Toronto at St. Michael's Hospital, Unity Health Toronto, Toronto, Ontario, Canada; c Department of Surgery, St. Michael's Hospital, Toronto, Ontario, Canada

**Keywords:** Pre-clerkship, Undergraduate medical education, Shadowing, Surgical workshops, Mentorship

## Abstract

**Introduction:**

Pre-clerkship medical students rely on various educational experiences to decide on the residency they would like to pursue. We conducted a longitudinal mixed-methods study to identify educational experiences in pre-clerkship that are associated with an interest in pursuing surgery.

**Methods:**

Pre-clerkship medical students were invited to complete an initial survey regarding their interest in surgery and educational exposures. After 10 months, a follow-up survey was sent to identify changes in their interest and the role of educational experiences they may have had in the interim. Univariate regression was used to determine associations, and thematic analysis was done.

**Results:**

Data from 218 pre-clerkship students showed that shadowing (OR = 2.7), participation in technical workshops (OR = 5.1), having a mentor (OR = 4.6) and conducting surgical research (OR = 4.6) were associated with an interest in pursuing surgery. From the students with follow-up data, thematic analysis showed that 94 %, 89 %, and 81 % of students found shadowing, research, and mentorship, respectively, as influential in the decision of pursuing a surgical specialty, respectively.

**Conclusions:**

Shadowing and mentorship were important factors for students in the decision-making process in pursuing surgery. Identifying high-yield educational experiences—for students to determine if one wants to pursue a surgical specialty is important for educators in curriculum design for resource allocation.

**Key message:**

We describe a longitudinal mixed-methods study to determine the role of early educational exposures which influence a medical student's decision to pursue a surgical specialty. Shadowing, technical skills workshops, surgical mentorship, involvement in surgical research, play an important role for student decisions.

## Introduction

Exposures to medical specialties play an important role in the professional development of early pre-clerkship students [1]. Pre-clerkship offers an opportunity to develop an interest in a student's specialty of choice by gathering educational exposures, via classroom teaching, workshops, research, formal and informal interactions with mentors [2,3]. However, most preclinical students are not offered a formal surgical exposure and it is not viewed as a crucial learning or exploratory practice [3]. Formal pre-clerkship surgical curriculums are uncommon in Canada. It is known that students garner interest in surgery from shadowing surgeons, having a surgical mentor, or being formally exposed to surgical subspecialty care [1]. Despite published literature exploring these activities that may potentially peak pre-clerkship student interest in surgery, no study to date has elucidate the narratives of students and their associated perceptions of these experiences—regarding high-yield activities.

Most of the research pays attention to demographics, preconceptions, personal relationships, and other predispositions that influence a pre-clerkship student's decision to pursue surgery [4]. Moreover, previous studies are often focused on individual subspecialties with specialty-specific experiences rather than examining surgical experiences broadly at either the institution or individual student level [[5], [6], [7]]. Altogether, it is accepted that an individual's decision about pursuing surgery are formed during pre-clerkship years, yet there is a knowledge gap on the strength of association between specific surgical exposures and the decision to pursue surgery [1]. Furthermore, there has yet to be published literature on student perception of both the utility of the experience and narrative (positive, negative, and neutral) of the activity.

Here, we performed a longitudinal mixed-methods study to quantify and qualify the role of pre-clerkship educational exposures in surgery with a student's interest in pursuing a residency in surgery. With this method, we aimed to quantify the importance of shadowing, surgical mentorship, research, technical skills workshops, classroom learning, and other experiences which affected decision-making in pre-clerkship students.

## Methods

### Setting, population, and study design

The study was conducted among medical students at the University of Toronto undergraduate MD program. The program entails 2 years of pre-clerkship with didactic learning and subsequently, 2 years of clerkship with direct clinical exposure. Within the pre-clerkship years, educational exposures are offered to all interested students, including technical skills workshops, career interest events, didactic lectures, among others. These exposures are a combination of student- and faculty-organized. A discussion among student organizers and faculty educators to determine the most relevant educational exposures for study was conducted before survey rollout.

After receiving ethics approval from the University of Toronto Research Ethics Board, pre-clerkship students in the University of Toronto undergraduate MD program were recruited via e-mail. The participants were sent an initial survey and follow-up survey using SurveyMonkey. The initial survey was sent in March 2021, and follow-up was conducted in January 2022. At the time of follow-up, the initial survey was sent to remaining eligible pre-clerkship students to enter the study without opportunity for follow-up.

### Data collection

The initial survey gauged student interest in pursuing surgical residency by asking whether students were “interested”, “not interested”, or “unsure” in pursuing surgical residency. The survey also captured which educational exposures each student had encountered. The surgical exposures included the following: lectures on surgical topics, surgical workshops, shadowing a surgeon, participating in the operating room, having a surgical mentor, and participating in surgical research (Supplementary Table 1), in addition to demographic information.

The follow-up survey again gauged any changes to each student's interest in pursuing surgery. Free-text questions (Supplementary Table 2) were provided for each student to describe which surgical exposures had an influence in their decision to pursue surgery. All data was kept anonymous and confidential with all participants being de-identified and data stored on a secured University of Toronto server.

### Statistical analysis

Descriptive statistics were tabulated on students stratified by interest in pursuing surgery. Univariate regression was conducted to determine the association between educational exposures and the decision to pursue surgical residency. For analytical purposes, participants who were “unsure” about pursuing surgery were pooled with “not interested” for dichotomous outcomes. Odds ratios (OR) and 95 % confidence intervals (CI) were calculated. All statistical analyses were performed using SPSS version 26 (IBM) and MatLab 2021a (MathWorks).

### Qualitative methods

From the results of the follow-up survey, we applied a thematic analysis to identify common patterns among the responses. Two researchers completed line-by-line coding of the data which were organized into categories and then into broader themes. The purpose of the thematic analysis is two-fold. The first is to elucidate student's perception of the utility of an activity in their decision making of whether to pursue surgery. The second is to further qualify that experience through the student narrative in the free text to determine the association of the activity with positive, neutral or negative attributes. Frequency perceptions and conceptions were used to determine influences on a student's decision to pursue residency in a surgical specialty. Special attention was placed on participants who were initially unsure about pursuing surgery and later changed their decision to either interested or not interested. All qualitative analysis was performed using Excel (Microsoft).

## Results

### Quantitative analysis

#### Baseline characteristics and interest in surgery

Of the 122 participants who completed the initial survey in March 2021, 81 completed the follow-up survey in January 2021 resulting in a retention rate of 66 %. At this time, 96 additional participants completed their initial survey which were pooled with the 122 participants, resulting in a total sample size of 218 for quantitative analysis with an estimated response rate of 29 %. The sample consisted of 139 females (64 %), 76 males (35 %), and 3 non-binary (1 %). Of the 218 students, 75 wanted to pursue surgery (34 %), 70 were unsure (32 %), and 73 did not want to pursue surgery (33 %) in their initial survey. Of the 81 students with follow-up, 61 were unchanged (75 %), 13 had reduced interest in surgery (16 %), and 7 had increased interest in surgery (9 %) at the time of follow-up.

To determine which educational exposures were associated with an initial interest in surgery, odds ratios were calculated (Table 1). Attending technical workshops, shadowing a surgeon, having a surgical mentor, and conducting research in surgery were all significantly associated (p < 0.05) with interest in surgery. While lectures on surgical topics and operating room exposure were not associated with an interest in surgery. Additionally, female sex was associated with a reduced odds of having interest in pursuing surgery (OR 0.42, 95 % CI 0.23, 0.75; p = 0.004).

**Table 1:** Baseline characteristics of study population and univariate regression.

	Not interested in surgery (n, %)	Interested in surgery (n, %)	Total sample (n, %)	Odds ratio (95 % CI); p-value
N	143	75	218	–
Male	40, 28 %	36, 48 %	76, 35 %	0.42 (0.23–0.75); p = 0.004
Female^a^	101, 71 %	38, 51 %	139, 64 %	
Non-binary	2, 1 %	1, 1 %	3, 1 %	
Surgical exposures				
Lectures on surgical topics and techniques	75, 52 %	40, 53 %	115, 53 %	1.03 (0.59–1.81); p = 0.90
Surgical or technical skills workshops	44, 31 %	52, 69 %	96, 44 %	5.09 (2.78–9.32); p < 0.001
Shadowing a surgeon	34, 24 %	34, 45 %	68, 31 %	2.66 (1.47–4.82); p = 0.001
Shadowing in the operating room	37, 26 %	28, 37 %	65, 30 %	1.71 (0.94–3.11); p = 0.08
Having a mentor who is a surgeon	26, 18 %	38, 51 %	64, 29 %	4.62 (2.48–8.60); p < 0.001
Participated or conducted research in surgery or a surgical subspecialty	19, 13 %	31, 41 %	50, 23 %	4.60 (2.36–8.96); p < 0.001

a Female sex was used as the positive exposure in the calculation of odds ratio.

### Qualitative analysis

#### Exploring the role of educational exposures in decision-making

The statistically significant factors were then explored qualitatively through a follow-up survey (Supplementary Table 1). Of the 81 participants providing us with follow-up data, 46 (57 %) participants commented on shadowing, 36 (44 %) participants commented on technical and surgical skills workshops, 41 (51 %) participants commented on mentorship and 18 (22 %) participants commented on research as having a relationship of influence in the decision to pursue surgery (Table 2). Shadowing was the educational exposure most associated with deciding to pursue surgery with 94 % of respondents stating that it is a deciding factor on pursuing surgery. This was followed by research (89 %), mentorship (81 %), and technical and surgical skills workshops (53 %). Interestingly, 22 % of participants who commented on technical and surgical skills workshops did not find them valuable tools to help them decide on surgical specialty, in contrast to a similar 25 % of participants who found those workshops ambiguous in their career decision making. A small minority (<10 %) in the remaining three categories of shadowing, research and mentorship did not find those activities compelling them one way or another towards a surgical career.

**Table 2:** Analysis of open responses to activity influencing decision to pursue surgery.

Influence of activity on decision to pursue surgery	Shadowing (n, %)	Surgical or technical skills workshops (n, %)	Mentorship (n, %)	Research (n, %)
Strong influence	43, 94 %	19, 53 %	33, 81 %	16, 89 %
Neutral influence	2, 4 %	9, 25 %	3, 7 %	–
No influence at all	1, 2 %	8, 22 %	5, 12 %	2, 11 %
Totals	46, 100 %	36, 100 %	41, 100 %	18, 100 %

#### High-yield activities in ambivalent students

Our study included 20 participants who were initially unsure on pursuing surgery. At follow-up, 14 (70 %) remained unsure, 4 decided not to pursue surgery (20 %), and 2 decided to pursue surgery (10 %). The participants who became interested suggest:

“*Shadowing first exposed me to surgery but I was not actively interested until I was taking part in [direct clinical exposure]*”

Participant 01-0034

“*More shadowing experiences that are easily accessible and mentorship opportunities with staff and residents [are needed].*”

Participant 01-0102

While those participants who became less interested were less likely to attend any educational exposures during the interim period. Of the 4 who decided not to pursue surgery, 2 had not attended or received any educational exposures while the other 2 had negative experiences shadowing.

#### Association of narrative of experience with educational exposures

The student's attribution of whether each education experience positively or negatively influenced their decision to pursue surgery was analyzed and categorized (Table 3). Examples of responses that were categorized as positive experiences include quotations such as “*Watching the surgical team work together in the OR [operating room] on multiple complex operations has made me more inclined to pursue surgery*” (Participant 01-0070) and “*Rewarding aspect of immediately making a difference for the patient.*” (Participant 01-0041). Examples of responses that were categorized with a negative narrative include “*Poor experience, surgeon was not receptive to answering questions and pretended I wasn't in the room while [they] gave all the attention to the other student*” (Participant 01-0084) and “*Staff that makes for poor and toxic learning environments (doctors and nurses alike) contribute to experiences swaying away from pursuing surgery*” (Participant 01-0071).

**Table 3:** Analysis of open responses to positive, neutral or negative attributes of activities.

Attribution	Shadowing (n, %)	Surgical or technical skills workshops (n, %)	Mentorship (n, %)	Research (n, %)
Positive experience	7, 41 %	5, 50 %	12, 52 %	–
Neutral experience	2, 12 %	4, 40 %	7, 30 %	–
Negative experience	8, 47 %	1, 10 %	4, 17 %	–
Totals	17, 100 %	10, 100 %	23, 100 %	0, −

Of the 46 participants who discussed shadowing as an influence, 17 participants in the narratives could be categorized (7 positive, 2 neutral, 8 negative).

“*It's the only way to get to know what a day in the life looks like and to understand how teams work together.*”

Participant 01-0127 [Positive influence]

“*Being in the [operating room] environment and seeing the team work together swayed me to pursue surgery; preceptors allowing me to do stuff in the [operating room] has also swayed me towards pursuing surgery*”

Participant 01-0008 [Positive influence]

Meanwhile, 10 participants discussed technical skills workshops as an important influence on pursuing surgery (5 positive, 4 neutral, 1 negative).

“*Learning about different suturing techniques/skills and seeing the complexity involved in mastering these skills has made me more interested in surgery*”

Participant 01-0070 [Positive influence]

“*Attending a suturing workshop was helpful in helping me be more comfortable with working with my hands.*”

Participant 01-0092 [Neutral influence]

“*…they are only helpful in acquiring skills but actually seeing the application to a patient in an [operating room] is key.*”

Participant 01-0091 [Negative influence]

Moreover, 23 participants placed importance on mentorship as an importance influence on whether to pursue surgery (12 positive, 7 neutral, 4 negative).

“*Having mentors in surgery and seeing how they model their life has really shown me the various options for what life could look like for me personally. My mentors have also been able to tell me about the good and bad things in their specialty without making it seem like they are trying to turn me away from it.*”

Participant 01-0025 [Positive influence]

“*Mentors from other fields perhaps pushed me away from surgery a bit through discussions about the importance of work-life balance in a career.*”

Participant 01-0109 [Negative influence]

Lastly, no participants expressed any attributable narrative in research.

## Discussion

Pre-clerkship is a critical period for medical students in their decision to pursue surgical residency. Our study identifies educational exposures which students perceive as important in their process of choosing surgery and quantifies their association with an interest in surgery. Overall, we show that shadowing, technical skills and workshops, and mentorship play a significant role in this important choice for students and participation in these exposures are associated with an interest in surgery. Furthermore, we were able to identify student's attributed narrative perception of those activities and quantify them. Together, this provides a framework for educators to identify “high yield” exposures as well as targets of improvement to ensure ideal nurturing of future surgeons and educating pre-clerkship students.

### Shadowing

Shadowing has been shown to increase student's understanding of routine practice in surgical specialty, beyond conventional lectures [8]. In other words, this exposure permits students to “test drive a specialty” and is analogous to a “screening tool” for students [9]. The most impactful activity for pre-clerkship students in the decision to pursue surgery was shadowing, where 94 % of students found this to be essential. Our thematic analysis showed shadowing is not inherently associated with a positive interest in surgery. Rather, students commented on the operating room environment, impact on family planning, and surgical culture in either a positive or negative light, with few participants feeling both. However, it is important to note that student's perception and comments on the activity post-shadowing were polarizing. It is not yet clear as to the why this experience, in comparison to the others, was more polarizing.

### Surgical and technical workshops

Workshops and technical programs have been developed at many institutions to help develop technical skills in pre-clerkship students before their surgical rotations [6,10]. We found that these were associated with an interest in surgery; however, we note that these experiences provided skills without providing a comprehensive lens into surgical practice. There may be a more significant role for subspecialty-focused workshops, attracting students with already more defined interest in surgery than general workshops, in definitively creating student interest in specialties than general technical workshops [[11], [12], [13]]. Moreover, these workshops were not very high-yield activities for pre-clerkship students who are undecided in the decision-making process. Finally, such workshops are often expensive and require a significant amount of planning and coordination.

### Surgical mentorship

Mentorship is well integrated into medical education and is known to be an important factor in deciding on any specialty [14,15] and especially for underrepresented groups [1]. Our study affirms that having a surgical mentor is strongly associated with an interest in surgery. Our thematic analysis showed that mentors can provide a holistic lens into a surgical career which other educational exposures may not be able to provide.

### Limitations

This study should be interpreted considering several limitations. First, this study includes students from a single institution with limited follow-up which limits the generalizability of the findings. Moreover, our data is limited by self-reported nature of our primary outcome (interest in surgery), with insufficient follow-up to validate the number of students who objectively chose to pursue a surgical residency. Next, we are unable to decouple which educational exposures are associated with a pre-existing versus newfound interest in surgery. This limits the applicability of our findings in determining new curricular developments to promote exploration for surgery in undergraduate medical education. Importantly, our study had limited follow-up and although all attempts were made to encourage retention, our findings are limited by small sample size with complete data. Furthermore, as with any qualitative research method, our findings are subject to interpretation bias and lead question bias since the questions were defined based on known associations. Lastly, this study was conducted during the COVID-19 pandemic which has greatly affected access to surgical exposures and the nature of them, respectively [16].

## Conclusions

Interest in surgery is associated with having experienced the following educational exposures: surgical or technical skills workshops, shadowing a surgeon, having a surgical mentor and performing research in surgery. Our study was the first of its kind to also include a narrative analysis which demonstrated that there are stark differences between perception of activities with students generally having a positive influence from mentorship while shadowing may have either positive or negative influence towards pursuing surgery. Further research is required to qualify the discrepancy between experiences and explore and inter-school differences.

## Funding

Funding was provided from the 10.13039/501100003579University of Toronto.

## Ethics approval

This study was approved by the University of Toronto Research Ethics Board.

## CRediT authorship contribution statement

AK, MHL, and EK carried out the surveys, quantitative, and qualitative analysis. JSS supervised this work. All authors contributed to the conceptualization of this work, writing of the manuscript, and editing of the manuscript.

## Conflict of interest

The authors have no relevant conflicts of interest to disclose.

## Appendix A Supplementary data

Supplementary Tables: Including survey format for initial and follow-up. Image 1
