# Supplementary material for: Educational exposures associated with preclinical medical student interest in pursuing surgical residency: Longitudinal mixed-methods study with narrative evaluation
Source: Surg Open Sci. 2023 Mar 3;12:43–7. doi: 10.1016/j.sopen.2023.03.002 (PMC10017410; doi:10.1016/j.sopen.2023.03.002)
Supplement: Supplementary file 1 — Supplementary Tables: Including survey format for initial and follow-up. [file mmc1.docx]

Supplementary Table 1: Initial survey from March 2021 and for new recruitment without follow-up in January 2022

| **Initial Survey** |
| --- |
| Year of study: ___ |
| Gender: ___ |
| Are you currently interested in pursuing surgery |
| **Yes No Unsure** |
| If yes or no, do you think your decision will change over the course of medical school? |
| **Yes No Unsure** |
| Have you had lectures on surgical topics and techniques? |
| **Yes No** |
| Have you participated in surgical or technical skills workshops? |
| **Yes No** |
| Have you shadowed a surgeon? |
| **Yes No** |
| Have you shadowed in the operating room? |
| **Yes No** |
| Do you have a mentor who is a surgeon |
| **Yes No** |
| Have you participated or conducted research in surgery or a surgical subspecialty? |
| **Yes No** |

Supplementary Table 2: Follow-up survey for eligible participants in January 2022

| **Follow-up Survey** |
| --- |
| Year of study: ___ |
| Which educational experiences have affected your decision to pursue surgery in pre-clerkship? |
| How has shadowing in surgery or otherwise influenced your decision? While shadowing,  have any experiences swayed you towards or away from pursuing surgery? |
| Have surgical skills workshops influenced your decision to pursue surgery and how? |
| Has mentorship or research in surgery influenced your decision to pursue surgery and how? |
| What further experiences should the Department of Surgery provide to help medical  students decide on whether or not to pursue surgery in pre-clerkship? |
